# The role of vitamin D in the link between physical frailty and cognitive function: A mediation analysis in community-dwelling Chinese older adults

**DOI:** 10.3389/fnut.2022.922673

**Published:** 2022-07-22

**Authors:** Jian Xiong, Wen-Xiong Xue

**Affiliations:** Department of Rehabilitation, Affiliated Zhangjiagang Hospital of Soochow University, Suzhou, China

**Keywords:** vitamin D, physical frailty, cognitive function, mediation analysis, older adults

## Abstract

**Background:**

Physical frailty and cognitive aging have important influences on poor clinical outcomes in older adults. Many studies have investigated the association between frailty and cognitive function, but whether vitamin D mediates the association between frailty and cognitive function is unclear. We explored the mediating role of vitamin D on the cross-sectional association between physical frailty and cognitive function using data from the Chinese Longitudinal Healthy Longevity Survey (CLHLS).

**Methods:**

We analyzed data from 1944 subjects aged 60 years and older from the 2011 CLHLS cohort. Frailty status was identified by the Osteoporotic Fracture Study (SOF) index. The Chinese version of the Mini-Mental State Examination (MMSE) was used to assess cognitive function. Linear regression models were used to examine the association between frailty, vitamin D, and cognition, adjusted for a range of covariates. Mediation analyses tested the indirect effects of vitamin D on physical frailty and cognitive function.

**Result:**

Physical frailty was negatively associated with vitamin D levels and scores on the MMSE, and vitamin D levels were positively associated with scores on the MMSE. Linear regression analysis showed that physical frailty and serum vitamin D concentration were significant predictors of cognitive function. Importantly, mediation analysis showed that serum vitamin D concentration significantly mediated the relationship between physical frailty and cognitive function.

**Conclusion:**

The association between physical frailty and cognitive function appears to be mediated by vitamin D. Future studies should explore whether serum vitamin D concentrations may mediate the association between physical frailty and cognitive decline and whether this mediating role is moderated by other factors.

## Introduction

Population aging continues to accelerate globally, especially in China, due to advances in health-care, public health, and social and economic development leading to increased survival rates. Between 2018 and 2050, China's population of people aged 60 and older will increase from 249 million to 478 million and from 17.9 to 35.1% of the total population (1). As the population of older adults increases, research hotspots and clinical practice are beginning to focus on physical frailty (PF), cognitive function, and their effects on functional independence in older adults (2). PF is a key intermediate state, which is a state of increased vulnerability and decreased responsiveness to stress in the aging process and is associated with multiple physiological systems gradually losing their intrinsic reserves (3–5). Although PF is an important predictor of some poor clinical outcomes, such as falls, dependence, hospitalization, disability, and death, (6) fortunately, it may be reversed or diminished by intervention (7). Another hallmark of neurological aging, cognitive aging, is characterized by deteriorating memory and reduced mental capacity and in turn leads to the development and progression of neuron-degenerative diseases (8). Although the age-related cognitive decline is a subtle and normative developmental process, it can damage many higher cortical functions, such as attention and executive function (9, 10), even increasing the risk of mild cognitive impairment and AD, and is becoming a public health concern (11, 12) Hence, PF and age-related cognitive decline directly affect physical health, increase disability, reduce the quality of life and lead to adverse consequences.

As aging is associated with physical frailty and cognitive decline, it is reasonable to understand the relationship between cognition and physical frailty. A large number of studies have verified that older adults with PF perform worse on global cognition (13, 14) and are more likely to experience cognitive decline(15–19) and cognitive impairment (20–22). In addition, there is also evidence that cognitive function can also have an impact on frailty in older people (23–25). However, few studies have explored the specific mechanisms of frailty and cognitive function. Therefore, to help maintain or improve the independent functioning and quality of life of older people, there is a need to further understand the possible mechanisms between physical frailty and cognitive function. Vitamin D, mainly synthesized in the skin during exposure to sunlight, also known as the “sunshine vitamin” and the “rickets vitamin,” is a group of fat-soluble vitamins that have a role in bone and muscle health, cardiovascular disease, and even mortality (26). Although the results of some studies have not found a clear relationship between 25(OH)D and the risk of frailty (27, 28), in a large number of other studies, it can be found that older people with lower 25(OH)D levels are more likely to be frail than those with higher 25(OH)D levels (29–32). Additionally, the results of several meta-analyses also indicated that lower 25(OH)D levels were significantly associated with an increase in the severity of frailty (33–35). High levels of serum vitamin D may protect against physical frailty and reduce its occurrence (34). In addition, vitamin D (25(OH)D3), a neurosteroid hormone required for normal brain regulation and development, is strongly associated with cognitive decline and neuron-degenerative diseases (36, 37). Epidemiological evidence has identified vitamin D as a valid predictor of cognitive decline or dementia in older people (38–40). In a population-based longitudinal study, older adults with low baseline vitamin D levels were significantly associated with cognitive decline as assessed by the Brief Mental Status Examination at 2 years (36) and even with the risk of developing Alzheimer's disease (41). Previous studies have focused too much on the relationship between physical frailty and cognitive function and have rarely explored the underlying mechanisms or potential mediating factors of this association; additionally, it has seldom been explored in large epidemiological cohorts of older people. Furthermore, there is no consensus on the results of the studies available to determine the exact relationship between vitamin D and frailty and cognitive function.

Therefore, using nationally representative longitudinal survey data, the current study aimed to examine the relationship between vitamin levels, frailty status, and cognitive function among community-dwelling older adults, as well as to verify the mediating role of vitamin levels between PF and cognitive function.

## Methods

### Participants

Participants were recruited from the 6th (2011) wave of CLHLS, which was the first and largest national, community-based, longitudinal prospective cohort survey. The samples were randomly selected from half of the 22 counties and municipalities of the 31 provinces that make up approximately 85% of the population of China. The sampling characteristics were as follows: for each centenarian who voluntarily agreed to participate in the study, one octogenarian and one non-agenarian of predefined age and sex were randomly selected and interviewed by the CLHLS, and for every two centenarians, three nearby people aged approximately 65-79 were randomly selected. The CLHLS collected biomarkers in the longevity regions, including Xiayi County in Henan Province, Zhongxiang City in Hubei Province, Yongfu County in Guangxi Autonomous Area, Laizhou City in Shandong Province, Sanshui District in Guangdong Province, Mayang County in Hunan Province, Chengmai County in Hainan Province and Rudong County in Jiangsu Province. Therefore, it provides information on basic demographics, health status, socioeconomic characteristics, and lifestyle of the elderly (42), in addition to collecting biomarker datasets for 30 indicators such as routine blood tests, blood biochemical tests, and urine tests (43).

More details of the CLHLS, such as the sampling design and assessment of data quality, are described at http://www.icpsr.umich.edu/icpsrweb/NACDA/studies/36179.

A total of 2,429 participants were initially enrolled in the study. In the analysis, younger ages (<60 years, *n* = 16) and missing data (cognitive function (*n* = 143), SOF index components (*n* = 116), Vitamin D3 (*n* = 91), and potential confounding variables (*n* = 129)) were excluded. Finally, we ultimately retained 1,944 older adults in this study. The study was approved by the Biomedical Ethics Committee of Peking University and Duke University, and all participants signed written informed consent forms.

### Measurement

#### Assessment of physical frailty

The current study relied on the Study of Osteoporotic Fractures (SOF) frailty index to define physical frailty, which includes three simple self-reported components: underweight (defined as body mass index<18.5), low energy level (indicated by a positive response to the question “Over the last 6 months, have you been limited in activities because of a health problem?”), and muscle strength (inability to stand up from a chair without the assistance of arms) (44). The SOF frailty index is considered to be a useful tool in assessing the physical aspects of frailty at the population level (44–46), and frailty determined by this method is associated with falls, disability, fractures, and death (47, 48). As suggested, participants with two or more of the three components were defined as frail.

#### Assessment of cognitive function

Cognitive function of the CLHLS participants was measured using the Chinese version of the Mini-Mental State Examination (MMSE), which measures four aspects of cognitive function: orientation, short memory, attention and computation, recall, and language, with scores ranging from 0 to 30 (49). To truly reflect the cultural and socioeconomic conditions of the elderly in China, a Chinese version of the MMSE was created, which was adapted from the international MMSE questionnaire and has been verified in previous studies (50). The score on the MMSE is recognized as continuous data, with lower scores indicating poorer cognitive function (51).

#### Assessment of serum 25(OH)D

Because serum 25(OH)D reflects the source of vitamin D from sunlight exposure and diet, it is considered the best biomarker of vitamin D status. Therefore, we measured serum 25(OH)D concentrations to represent vitamin D levels(52). A detailed description of how to collect fasting venous blood and collect and transport blood samples has been published elsewhere (53). Plasma 25-hydroxyvitamin D [25(OH)D] levels were measured using an enzyme-linked immunosorbent assay (Immunodiagnostic Systems Limited, Bolton, UK), and the inter- and intra-assay coefficients of variation were <10 and 8%, respectively. The measured result was expressed in nmol/L.

#### Covariates

The covariates adjusted for in this study, including sociodemographic variables, health condition information, and confounding biomarkers, were obtained through structured questionnaires, physical examinations, and biomarker collections.

Sociodemographic variables included age, sex (female/male), marital status (married/other), place of residence (rural/other), and years of schooling.

Health status information is obtained through self-reporting and includes lifestyle and chronic disease status. The former included smoking (yes/no), alcohol consumption (yes/no), and exercise (yes/no), and the latter consisted of hypertension (yes/no), diabetes (yes/no), heart disease (yes/no), cerebrovascular disease (yes/no), and respiratory disease (yes/no). Hypertension was diagnosed by systolic blood pressure ≥ 140 mmHg and/or diastolic blood pressure ≥90 mmHg (54). Diabetes mellitus was defined as fasting plasma glucose ≥7.0 mmol/L (36) Residual disease was identified by self-report.

The confounding biomarkers included indicators from both routine blood tests and blood biochemical tests and were performed by the central clinical laboratory at Capital Medical University in Beijing. The assessment of blood samples analysis was determined by using a commercial diagnostic kit (Roche Diagnostics, Germany) on an automated biochemistry machine (Hitachi 7180, Japan). Specifically, the Immunoturbidimetric assay method was used to measure C-reactive proteins (CRPS), the Cholesterol oxidase method was used to measure total cholesterol (CHO), and the picric acid method was used to test serum creatinine (CREA). The glycerol phosphate oxidase-peroxidase method was used to measure triglyceride (TG), high-density lipoprotein cholesterol (HDL-C) by the direct method, and low-density lipoprotein cholesterol (LDL-C) by the Friedewald formula [LDL-C= TC-HDL-C-TG/2.17 (in mmol/L)]. Furthermore, the thiobarbituric acid method was used to measure malondialdehyde (MDA) and the xanthine/xanthine oxidase method was used to assay superoxide dismutase (SOD).

#### Statistical analysis

First, numbers and percentages were used to describe categorical variables, while means (standard deviation, SD) or medians (interquartile range, IQR) were used to describe continuous data. ANOVA, Kruskal–Wallis test, or χ2 test were used to compare characteristics among groups.

Second, Spearman's coefficient was used to test the correlation between the variables.

**Figure 1:**
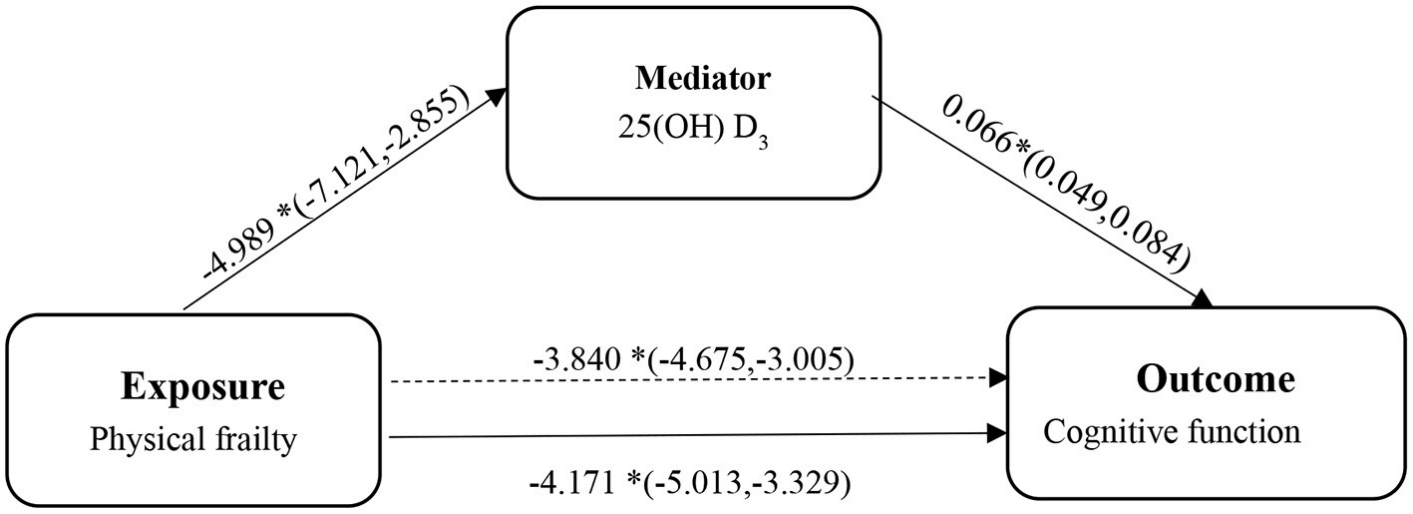
The diagram of the mediation analysis of physical frailty on cognitive function. ^*^p < 0.05.

Third, to determine the possible mediator role of vitamin D between physical frailty and cognitive function, we designed a mediation analysis (see Figure 1). We conducted the following analysis based on the multiple linear regression method technique proposed by Baron and Kenny (55): (1) exploring the relationship between cognitive function and physical frailty; (2) estimating the correlation between vitamin D and physical frailty; and (3) exploring the relationship between cognitive function and physical frailty following the incorporation of vitamin D. All analyses were adjusted for covariates. Finally, bootstrapping was used to assess the significance of the overall, indirect and direct utility of the mediation model. In the mediation analysis model, all paths were reported as unstandardized ordinary least squares regression coefficients, namely, total effect of X on Y (c) = indirect effect of X on Y through M (a × b) + direct effect of X on Y (c'). All statistical analyses were performed using SPSS version 21.0 (IBM, Armonk NY, USA) and the mediation effect was conducted in the Model 4 of PROCESS INDIRECT Macro 3.4. A *p*-value <0.05 was considered statistically significant. The 95% Cl for direct effects or indirect effects was based on a self-help sample of 5,000 and was considered statistically significant if the 95% Cl excluded zero.

## Result

### Participant characteristics

Table 1 shows the characteristics of the participants. Among 1,944 older adults, the mean age was 85.06 (standard deviation: 2.75) years, and 46.8% were male. Among these participants, the prevalence of PF was 22.8%. Those who were physically frail were more likely to be older and female than those who were not physically frail. The mean values of 25(OH)D3, total cholesterol, low-density lipoprotein cholesterol, and triglycerides and the number of years of education for subjects who were physically frail were significantly lower than those for non-physically frail subjects, while C-reactive proteins and superoxide dismutase were significantly higher. Smoking, drinking, and outdoor activities were more common in cognitively intact participants. The prevalence of at least one ADL limitation was higher in cognitively impaired subjects than in cognitively intact subjects. Being married, living in rural areas, smoking, drinking, exercising, diabetes, cerebrovascular diseases and respiratory diseases were more common among participants who were not physically frail. The mean score for MMSE of the frail subjects was significantly lower than that of the frail subjects.

**Table 1:** Sample characteristics according to periodontal status.

**Variables**	**Overall (*****n*** = **1949)**	**Physical frailty status**		***P*** **value**
		**Non-PF (*****n*** = **1500)**	**PF (*****n*** = **444)**
**Sociodemographics, n(%)**				
Age,M (SD)	85.06 (12.75)	82.00 (12.04)	95.39 (9.14)	<0.001
Gender(male)	913 (46.8)	795 (52.9)	118 (26.5)	<0.001
Married	818 (42.0)	745 (49.6)	73 (16.4)	<0.001
Years of school	0 (0, 4)	0 (0, 5)	0 (0,0)	<0.001
Rural	1640 (84.4)	1266 (84.4)	374 (84.2)	0.933
**Health characteristics, *n* (%)**				
Current smoking	342 (17.5)	301 (20.0)	41 (9.2)	<0.001
Current drinking	327 (16.8)	295 (19.6)	32 (7.2)	<0.001
Current regular exercise	321 (16.5)	275 (18.3)	46 (10.3)	<0.001
Hypertension	518 (26.6)	419 (27.9)	99 (22.2)	0.058
Diabetes	43 (2.2)	35 (2.3)	8 (1.8)	0.028
Heart diseases	142 (7.3)	112 (7.5)	30 (6.7)	0.055
Cerebrovascular diseases	147 (7.5)	103 (6.9)	44 (9.9)	0.001
Respiratory diseases	172 (8.8)	128 (8.5)	44 (9.9)	0.012
**Biomarkers, *M* (IQR)**				
25 (OH) D (nmol/L)	39.55 (28.48, 54.10)	42.20 (30.89,56.47)	31.13 (23.30, 43.22)	<0.001
CRPS (mg/L)	0.89 (0.39, 2.45)	0.87 (0.39,2.25)	1.09 (0.41, 3.59)	0.004
Vitamin B_12_ (pmol/L)	346.50 (249.25, 500.00)	350.00 (256.00,503.00)	324.00 (229.50, 494.25)	0.237
CHO (mmol/L)	4.25 (3.60, 4.92)	4.27 (3.64,4.94)	4.10 (3.49, 4.76)	0.004
CREA (mmol/L)	77.00 (64.25, 92.00)	77.00 (65.25,92.00)	75.00 (61.00, 92.75)	0.739
HDLC (mmol/L)	1.24 (1.03, 1.50)	1.25 (1.03,1.50)	1.23 (1.04, 1.48)	0.900
LDLC (mmol/L)	2.5 (1.98, 3.04)	2.52 (2.00,3.06)	2.44 (1.89, 2.95)	0.039
TG (mmol/L)	0.82 (0.60, 1.17)	0.83 (0.60,1.21)	0.77 (0.59, 1.07)	<0.001
SOD (IU/mL)	57.77 (52.56, 62.56)	57.43 (52.11,62.22)	59.11 (53.96,63.54)	<0.001
MDA (umol/L)	4.80 (3.83, 5.86)	4.81 (3.87,5.89)	4.78 (3.68, 5.70)	0.757
Baseline MMSE score	28 (21,29)	28 (25, 29)	19.00 (6, 27)	<0.001

*PF, physical frailty; CRPS, C-reactive proteins; CHO, total cholesterol; CREA=serum creatinine; HDLC, high-density lipoprotein cholesterol; LDLC, low-density lipoprotein cholesterol; TG, triglycerides, MDA, malondialdehyde, and SOD, superoxide dismutase;81CMMSE, the Mini Mental State Examination*.

### Association between the level of 25(OH) D and the risk of frailty

Table 2 shows a significant negative association between the level of 25(OH) D and the risk of frailty. This association persisted even after adjusting for a range of covariates. In addition, in the final model, the level of 25(OH) D in the older adults without PF was 4.928 (2.777, 7.079) higher than that in those with PF.

**Table 2:** The associations between physical frailty and serum levels of 25(OH) D_3_ (nmol/L).

	**Model 1** ^a **^	**Model 2** ^b **^	**Model 3** ^c **^	**Model 4** ^d **^
PF	reference	reference	reference	reference
Non-PF	10.488 (8.477,12.499)	5.138 (2.988,7.287)	5.029 (2.878,7.179)	4.989 (2.855,7.122)

a *Unadjusted model, B (95% CI);*

b *Adjusted for sociodemographics (age, sex, marital status, years of school, residence, B (95% CI));*

c *Adjusted for sociodemographics (age, sex, marital status, years of school, residence, health characteristics (smoking, drinking, regular exercise, and chronic diseases (e.g., hypertension, diabetes, heart diseases, stroke or cerebrovascular diseases, and respiratory diseases, B (95% CI));*

d *Adjusted for sociodemographics (age, sex, marital status, years of school, residence, health characteristics (smoking, drinking, regular exercise, and chronic diseases (e.g., diabetes, stroke or cerebrovascular diseases, and respiratory diseases) and confounding biomarkers (CRPS, CHO, LDLC, TG, SOD), B (95% CI)))*,

** *P < 0.01*.

### Association between frailty and cognitive function

Table 3, the model without mediators (25(OH) D) showed that compared to non-PF subjects, frail subjects had significantly lower MMSE scores (−4.171, 95% CI = −5.013; −3.329). When including 25(OH) D in the model, even though the correlation was weakened, it was still statistically significant (−3.840, 95% CI = −4.675; −3.005).

**Table 3:** The associations between physical frailty and cognitive function.

	**Model 1** ^a **^	**Model 2** ^b **^	**Model 3** ^c **^	**Model 4** ^d **^
PF	reference	reference	reference	reference
Non-PF	8.728 (7.867, 9.590)	4.270 (3.419, 5.120)	4.235 (3.387, 5.082)	4.171 (3.329, 5.013)

a *Unadjusted model, B (95% CI);*

b *Adjusted for sociodemographics (age, sex, marital status, years of school, residence, B (95% CI));*

c *Adjusted for sociodemographics (age, sex, marital status, years of school, residence, health characteristics, (smoking, drinking, regular exercise, and chronic diseases) (e.g., hypertension, diabetes, heart diseases, stroke or cerebrovascular diseases, and respiratory diseases), B (95% CI));*

d *Adjusted for sociodemographics (age, sex, marital status, years of school, residence, health characteristics) (smoking, drinking, regular exercise, and chronic diseases) (e.g., diabetes, stroke or cerebrovascular diseases, and respiratory diseases) and confounding biomarkers (CRPS, CHO, LDLC, TG, SOD), B (95% CI)*,

** *P < 0.010*.

### Mediating effect of 25(OH) D on the association between cognitive function and frailty

We subsequently examined whether 25(OH) D mediated the relationship between frailty and cognitive function using the PROCESS macro for SPSS. As shown in the mediation model (see Figure 1), we found that the independent variable (physical frailty) had an inverse relationship with cognitive function (β = −4.171, 95% CI = −5.256; −3.087). In addition, we found that physical frailty was inversely associated with (25(OH) D) (β = −4.989, 95% CI = −7.121; −2.855). The indirect effects showed that (25(OH) D) (β = −0.331, 95% CI = −0.489; −0.195) was an independent mediator of the detrimental effect of physical frailty on cognitive function.

## Discussion

This is the first study to examine the possible mediating role of vitamin D on the relationship between physical frailty and cognitive function in a community elderly population in a long-lived region of China. We found a significant negative association between physical frailty and both vitamin D and cognitive function. In addition, our results suggest that the effect of frailty on cognitive function was partially mediated by vitamin D among the elderly community.

Although a number of studies have investigated the relationship between 25(OH) D levels and frailty, both cross-sectional surveys and longitudinal studies appear to have reached inconsistent conclusions. However, relevant meta-analyses have confirmed this association. One meta-analysis showed a significant association between low levels of 25(OH) D and the risk of frailty compared to high levels of 25(OH) D (33). Similarly, the results of another meta-analysis found that 25(OH) D concentrations were significantly lower in frail older people than in non-frail older people (35). As hypothesized by the study, the current findings suggest that even when frailty is identified using the SOF frailty index, levels of 25(OH) D concentrations are significantly lower in frail older adults than in non-frail older adults after adjusting for a range of covariates. Our findings are also consistent with previous studies involving older adults from Spain (56), the USA (32), Mexico (57), Italy (58), Germany (59), China (60), and the Netherlands (61). However, due to the lack of sufficient evidence to prove the effectiveness of vitamin D supplementation on elderly individuals with PF, it is necessary to further explore the role of vitamin D supplementation in the intervention treatment of elderly individuals with PF in the future.

In addition, certain factors have been identified in the literature as potential risk factors for frailty, such as chronic diseases, lifestyle, and some biomarkers (62–64).

However, after adjusting for these covariates in our study model, the association between 25(OH) D and frailty was not significantly confounded. This suggests that 25(OH) D is independently associated with the risk of frailty. Nevertheless, there are unknown factors that were not included in this study and whether these factors influence this relationship, so more comprehensive research is needed in the future.

The negative association between frailty and cognitive function has been well studied. A recent systematic review and meta-analysis of cross-sectional studies examining the relationship between physical frailty and cognitive function in older adults showed that frailty status had a significant negative effect on cognitive function, both in terms of overall cognitive function and in terms of individual cognitive domains. Furthermore, even after adjusting for age, the frailty assessment tool used and cognitive functioning status, the effect was not significantly reduced (65). Similarly, the results of this study indicate that frailty is significantly and negatively associated with cognitive function. Both physical frailty and cognitive decline are closely associated with aging, so both may have similar physiological mechanisms. Currently, available evidence suggests several mechanisms, such as increased proinflammatory states, mitochondrial dysfunction, epigenetic changes, hypothalamic-pituitary axis (HPA) dysfunction, AD pathology, hormones, nutrition, cardiovascular risk, mental health, and oxidative stress, that may be used to explain the link between physical frailty and cognitive function(24, 66, 67).

In this study, we found a positive association between the level of vitamin D and cognitive function, i.e., older adults with high levels of vitamin D concentrations in China were more likely to report higher MMSE scores than those with low levels of vitamin D concentrations. There is evidence of a protective effect of high levels of vitamin D concentrations on cognitive decline, dementia, and Alzheimer's disease in older adults (36, 41, 68, 69), which is consistent with our findings. The reason for this may be that vitamin D acts as a cognitive protector by controlling oxidative stress, inflammation, and energy metabolism through its own receptor VDR (70, 71), but these findings were conducted in preclinical studies, therefore, the improvement effect of vitamin D supplementation is contradictory at this stage. As only a few randomized controlled trials (RCTs) have explored the effect of vitamin D supplementation on cognitive performance in older people, only two RCTs identified an improvement in cognitive function by vitamin D supplementation, and participants had cognitive impairment (69, 72). Additionally, we also found that vitamin D significantly mediated the association between physical frailty and cognitive decline. More specifically, a high level of serum vitamin D concentrations may partially counteract the effects of physical frailty on cognitive decline, a key finding in large epidemiological cohorts of older people. This study provides preliminary evidence that vitamin D supplementation appears to improve cognitive function performance in people with physical frailty. In summary, through our findings, we suggest that early monitoring of vitamin D levels in community-dwelling older adults may be warranted for better healthy aging. Second, the results of this mediated effects analysis highlight the importance of physically frail patients for the inclusion of vitamin D and cognitive performance in clinical practice. In response to the current findings, geriatrics, nutrition departments, and allied health professionals should appropriately increase their attention to vitamin D levels in the clinical practice of interventions for frail patients to intervene in cognitive decline.

The strength of the current study is that plasma blood samples were collected from participants from multiple communities in multiple regions of China, which allowed us to increase the credibility of the samples and enable our study to produce reliable results. In addition, to our knowledge, this is the first time that the relationship between vitamin D in physical frailty and cognitive function has been explored nationally in older Chinese adults.

This study also has limitations that may affect our interpretation of the findings. First, the cross-sectional study design did not allow for an examination of the causal relationship between physical frailty and cognitive decline because of the lack of temporality. Specifically, the exposure and the outcome are measured at the same point in time, rather than before the outcome occurs. Second, cognitive function was assessed *via* the MMSE scale. Because of the ceiling effect of MMSE scores, the MMSE is not a substitute for a complete final clinical diagnosis evaluation of any individual. In addition, potential confounders in this data release were collected through self-reported formats, potentially introducing bias in the analysis. Finally, although this study controlled for preexisting confounders to the extent possible, the possibility of other potential confounders cannot be ruled out.

## Conclusions

In a cohort of older Chinese adults with representative longevity, frailty was found to be associated with significantly poorer cognitive performance, and frail older adults reported lower vitamin D levels. Furthermore, the association between frailty and impaired cognitive function appears to be mediated by vitamin D deficiency. Future studies should explore whether low vitamin D levels may mediate the association between physical frailty and cognitive decline and whether this mediating effect is regulated by oxidative stress, inflammation, and energy metabolism.

## Data availability statement

The original contributions presented in the study are included in the article/supplementary material, further inquiries can be directed to the corresponding author/s.

## Ethics statement

Written informed consent was obtained from the individual(s) for the publication of any potentially identifiable images or data included in this article.

## Author contributions

Writing—original draft preparation and writing—review and editing: JX. Project administration: W-XX. All authors contributed to the article and approved the submitted version.

## Conflict of interest

The authors declare that the research was conducted in the absence of any commercial or financial relationships that could be construed as a potential conflict of interest.

## Publisher's note

All claims expressed in this article are solely those of the authors and do not necessarily represent those of their affiliated organizations, or those of the publisher, the editors and the reviewers. Any product that may be evaluated in this article, or claim that may be made by its manufacturer, is not guaranteed or endorsed by the publisher.
